# Mediatisation and datafication in the global COVID-19 pandemic: on the urgency of data literacy

**DOI:** 10.1177/1329878X20947563

**Published:** 2021-02

**Authors:** Dennis Nguyen

**Affiliations:** HU University of Applied Sciences Utrecht, The Netherlands

**Keywords:** crisis, data literacy, datafication, digital transformation, mediatisation

## Abstract

In the COVID-19 pandemic, societal discourses and social interaction are subject to rapid mediatisation and digitalisation, which accelerate datafication. This indicates urgency for increasing data literacy: individual abilities in understanding and critically assessing datafication and its social implications. Immediate challenges concern misconceptions about the crisis, data misuses, widening (social) divides and (new) data biases. Citizens need to be on guard in respect to the crisis’ impact on the next stages of the digital transformation.

## Mediatisation and datafication in the crisis

Media play an essential role in how societies make sense of COVID-19 as a crisis of historical proportions (Bauman and Bordoni, 2014) and how they behave in this new context born of rapid disruption. I distinguish here between two general forms of media that shape societies’ actions (Couldry and Hepp, 2017): First, there are *media of public information*. These include news media, governmental/non-governmental channels, social media and so on (#COVID19). They serve for observing and influencing developments and events from different viewpoints. The political economy of news media, the role of digital platforms and questions of framing (-effects) are important subjects for critical analysis. Examples are racism and challenges of information disorder (dis-/misinformation, ‘fake news’, propaganda), as data-driven social media platforms and the algorithmic affordances of personalisation-focused systems shape the pandemic discourse.

Second, there are *media technologies for (social) interaction*. These enable remote interactions between individuals, organisations and non-human agents (Bunz and Meikle, 2018). As the majority accommodate their lives to lockdown-routines, the digital transformation accelerates. Video-calling, app-based services, social media, remote-work tools and streaming platforms are integral for a growing spectrum of activities: socialisation (e.g. family gatherings, parties and dating via Zoom); work, primarily in the service- and information sectors (e.g. Microsoft Teams); education, as universities offer hastily designed online curricula; entertainment, where streaming services further establish dominance; and consumption, as the online ordering of consumer goods increases. Social media serve all of these purposes and shape interactions between users through their affordances. They provide substitutes for physical spaces, which facilitates social life under lockdown conditions but simultaneously strengthens leading technology companies’ dominance in the digital economy. Accessibility of digital media and the extent of options for consumption vary between individuals and the regions they reside in, but even in ‘underdeveloped’ countries, digital technology is central to many people’s lives. These developments pre-date the crisis but intensified since its emergence.

In addition, there are new applications of governance, so-called ‘corona-apps’, that directly target a social root cause of the pandemic: the spread of infections through human contact. ‘Contact-trackers’ monitor infection paths/rates, which ideally allows for targeted quarantine measures. Presumably, this enables governments to gradually re-open public life. Several governments already use such apps (Singapore, China, Austria, South Korea) or are in the process of launching them (Netherlands, Germany, United Kingdom). Countries that have them report different results, and political-cultural factors influence citizens’ responses. While ‘early adopters’ such as South Korea see successes, the Singaporean variant was less warmly received. Governments usually supervise the process but private companies, most notably Google and Apple, offer independent alternatives. ‘Corona-apps’ raise important questions about privacy, data surveillance and data ownership, which can influence citizens’ willingness to use them. The Singaporean example shows that design and usability are important factors, too.

The prevalence of different digital media catalyses a far-reaching ‘mediatization’ (Couldry and Hepp, 2017) and digital transformation of diverse social and goal-oriented communication. It simultaneously intensifies the datafication of society. This in turn asks for critical reflection on the role of data in the understanding *of* and behaviour *in* the crisis context.

## Data – the ubiquitous element

In the pandemic, data are everywhere. Concerning *media of public information*, data serve for illustrating developments and assessing policies. Data visualisations are recurring framing devices for discussing the crisis’ diverse impacts. Media coverage relies heavily on statistics that provide quickly accessible overviews. Numbers appear to convey facts and imply accuracy through real-time monitoring; interactive maps and charts (‘flattening the curve’) dominate the ‘crisis-imagery’. It seems as if global society looks at the crisis through a data lens to understand and master it. Indeed, data drive digital solutions designed for various stakeholders to combat the virus (Corona Virus Tech Handbook, 2020).

The ubiquity of data raises some concerns. A recent study shows that media audiences may not understand the differences between logarithmic and linear scales (LSE Blog, 2020). This can contribute to misconceptions about the pandemic’s magnitude. Another complex problem is that despite vast amounts of data, which imply predictability, there is an inevitably strong notion of uncertainty about the crisis and the turns it may take. This uncertainty also derives from a loss of trust in public authorities and polarisation in public discourses (Miller, 2020). Uncertainty drives emotional and behavioural responses; it is cause *for* and result *of* conflicts between different (ideological) views on the pandemic. Data do not automatically facilitate consensus-finding but become object of disputes themselves, which can lead to more uncertainty. The crisis highlights that data alone cannot immediately solve hands-on health challenges, which are often a result of societal frictions (e.g. underfunded healthcare sectors). There are also technical issues: data can mislead and need critical assessment against their specific background. Especially concerning corona-apps, misplaced confidence in accuracy via datafication may lead to blindness towards issues related to measurement, analysis and subsequent interpretation (e.g. false positives, biased data).

Additional risks emerge due to data that we create because of the crisis via *media technologies for (social) interaction*. The extent of datafication is likely to increase considerably with a new societal model emerging that aims for maximum possible remoteness. Novel data may become harvestable as users repurpose digital solutions in creative ways. Video-calling as a means for performing cultural rituals (e.g. baptisms) is one example, using virtual reality for remote poker matches another (NYT Online, 2020). While this offers opportunities for tech companies, it can also increase users’ vulnerabilities to surveillance and cybercrime.

## The urgency for data literacy

There are multiple risks that decision-makers, solution-providers and citizens need to address critically and transparently: (1) technical issues of flawed, incomplete and biased data that can lead to inaccurate conclusions – especially in regard to digital tools for ‘pandemic control’; (2) low data literacy among larger parts of the public; (3) opportunities for powerful organisations to expand their influence and/or to enter even more domains of the public-private spheres; (4) data ownership and ethics; and (5) data freedom, security and protection. Data literacy offers paths to holistic strategies for addressing these challenges. In short, data literacy includes skills in reading and interpreting data, critical thinking but also understanding the implications of datafication through mediatisation. It is a cornerstone of digital media literacy, a tool of empowerment and shapes social practices (Engebretsen and Kennedy, 2020). It is an important factor for retaining agency and enabling inclusion as well as resistance in the digital society.

Data literacy increases resilience against harmful effects of intended and unintended malpractices. Defining harmfulness/undesirability is a cultural and/or political question; but acknowledging the urgency of data literacy and finding consensus about critical data issues is nevertheless crucial for navigating a transformational period. It is important to ensure transparency and open the ‘black boxes of algorithmic decision-making’ (Prinsloo, 2020). However, access and openness are not universal solutions (Obar, 2020). Data are complex issues and lay people need support and guidance in understanding what data mean for them and others (Obar, 2020). That is not to say that citizens have no clear expectations towards datafication. To the contrary, recent findings imply that users want more control and accountability (Hartman et al., 2020); but experts need to listen closer to these demands and engage in dialogues with the public about perceived risks.

While questions of data privacy are important, the potential societal impact of unchecked datafication go much further and concern different social biases. Overconfidence in data-driven technology bears risk to ignore their limitations, especially in regard to the deeper historical roots of the challenges that most countries face. Loukissas (2019) reminds us that ‘all data are local’; and localities have their very own social conflict constellations. Social injustice in the pandemic is a real threat that needs consideration in regard to the meaning, role and value of data. What is datafied to which end? Who is affected, who benefits and who faces disadvantages? The main challenge is to find ways in which data help solving problems while maintaining inclusion and fairness. These questions need immediate attention as there are already various incidents of data abuse/misuse via tracking apps: for example, during the recent protest wave in the United States, police forces used ‘corona-apps’ for monitoring protesters’ movements. In South Korea, data from similar apps contributed to the discrimination and stereotyping primarily of the LGBTQI+ community.

The argument for data literacy must become more prominent over the next phases of the pandemic, which are likely to intensify datafication. Increasing data literacy is an interdisciplinary effort on several fronts. Educators and researchers (especially in Internet studies, critical algorithm studies, media psychology, communication studies and media design) play a key role in identifying problems and initiating discussions that aim for viable and valuable solutions: diagnostically, that is, through observation and description of problems; prognostically, that is, through indicating causes and outlining consequences; and didactically, that is, through proposing normative-ethical countermeasures.

For *media of public information*, we need a better understanding of how public discourses conceptualise and apply data for explaining the crisis and proposing ways forward. This is important for opinion formation and individual empowerment. Understanding the algorithmic component in agenda-setting on social media also connects directly to data literacy. Concerning *media of (social) interaction*, we need to gauge the extended datafication of socialisation in various contexts and keep a watchful eye on how this trend may increase individual vulnerabilities. ‘Corona-apps’ raise questions about data ownership and alternative uses, as they add another layer to digital identity. How open societies can build transparent systems for monitoring and governing data uses is a key question.

Data influence how societies make sense of, try to overcome and behave in crisis. The pandemic is datafied and datafies simultaneously. Given the general stage of digital transformation in human history, this is little surprising. And despite the call for caution above, data-driven approaches have the potential to alleviate pressures and increase efficiency in many domains. Data are essential for finding orientation in highly complex and dynamic situations. It is not my intention to generally reject data as a source of understanding and tool for solution development but to call for more critical assessment of datafication both as a strategy a*gainst* and as a result *of* the crisis. The pandemic marks a watershed moment with implications of enormous complexity. The challenges above are central to the transformations that are already happening, and all stakeholders need to respond with adequate vigilance.

## References

[bibr1-1329878X20947563] Bauman Z Bordoni C (2014) State of Crisis. London: Polity Press.

[bibr2-1329878X20947563] Bunz M Meikle G (2018) The Internet of Things. London: Polity Press.

[bibr3-1329878X20947563] Corona Virus Tech Handbook (2020) Available at: https://coronavirustechhandbook.com/contents (accessed 23 May 2020).

[bibr4-1329878X20947563] Couldry N Hepp A (2017) The Mediated Construction of Reality. London: Polity Press.

[bibr5-1329878X20947563] Engebretsen M Kennedy H (eds) (2020) Data Visualization in Society. Amsterdam: Amsterdam University Press.

[bibr6-1329878X20947563] Hartman T Kennedy H Steedman R , et al. (2020) Public perceptions of good data management: findings from a UK-based survey. Big Data & Society. Epub ahead of print 22 June. DOI: 10.1177/2053951720935616.

[bibr7-1329878X20947563] Loukissas YA (2019) All Data Are Local. Cambridge, MA: MIT Press.

[bibr8-1329878X20947563] LSE Blog (2020) The public do not understand logarithmic graphs used to portray COVID-19. Available at: https://blogs.lse.ac.uk/covid19/2020/05/19/the-public-doesnt-understand-logarithmic-graphs-often-used-to-portray-covid-19/?utm_campaign=Blogs&utm_content=1590039120&utm_medium=social&utm_source=linkedin (accessed 23 May 2020).

[bibr9-1329878X20947563] Miller V (2020) Understanding Digital Culture, 2nd edn. London: Sage.

[bibr10-1329878X20947563] NYT Online (2020) The coronavirus crisis is showing us how to live online. Available at: https://www.nytimes.com/2020/03/17/technology/coronavirus-how-to-live-online.html (accessed 10 July 2020).

[bibr11-1329878X20947563] Obar JA (2020) Sunlight alone is not a disinfectant: consent and the futility of opening Big Data black boxes (without assistance). Big Data & Society 7(1): 205395172093561.

[bibr12-1329878X20947563] Prinsloo P (2020) Of ‘black boxes’ and algorithmic decision-making in (higher) education – a commentary. Big Data & Society. Epub ahead of print 22 June. DOI: 10.1177/2053951720933994.

